# Evaluation of human dermal fibroblasts directly reprogrammed to adipocyte-like cells as a metabolic disease model

**DOI:** 10.1242/dmm.030981

**Published:** 2017-12-01

**Authors:** Jian-Hua Chen, Kim Jee Goh, Nuno Rocha, Matthijs P. Groeneveld, Marina Minic, Timothy G. Barrett, David Savage, Robert K. Semple

**Affiliations:** 1 The University of Cambridge Metabolic Research Laboratories, Wellcome Trust-MRC Institute of Metabolic Science, Cambridge, CB2 0QQ, UK; 2 The National Institute for Health Research Cambridge Biomedical Research Centre, Cambridge, CB2 0QQ, UK; 3 The Medical School, University of Birmingham, Birmingham, B15 2TT, UK

**Keywords:** Fibroblasts, Adipocytes, Lipodystrophy, Reprogramming, Insulin resistance

## Abstract

Adipose tissue is the primary tissue affected in most single gene forms of severe insulin resistance, and growing evidence has implicated it as a site at which many risk alleles for insulin resistance identified in population-wide studies might exert their effect. There is thus increasing need for human adipocyte models in which to interrogate the function of known and emerging genetic risk variants. However, primary adipocyte cultures, existing immortalised cell lines and stem-cell based models all have significant biological or practical limitations. In an attempt to widen the repertoire of human cell models in which to study adipocyte-autonomous effects of relevant human genetic variants, we have undertaken direct reprogramming of skin fibroblasts to adipocyte-like cells by employing an inducible recombinant lentivirus overexpressing the master adipogenic transcription factor PPARγ2. Doxycycline-driven expression of PPARγ2 and adipogenic culture conditions converted dermal fibroblasts into triglyceride-laden cells within days. The resulting cells recapitulated most of the crucial aspects of adipocyte biology *in vivo*, including the expression of mature adipocyte markers, secreted high levels of the adipokine adiponectin, and underwent lipolysis when treated with isoproterenol/3-isobutyl-1-methylxanthine (IBMX). They did not, however, exhibit insulin-inducible glucose uptake, and withdrawal of doxycycline produced rapid delipidation and loss of adipogenic markers. This protocol was applied successfully to a panel of skin cells from individuals with monogenic severe insulin resistance; however, surprisingly, even cell lines harbouring mutations causing severe, generalised lipodystrophy accumulated large lipid droplets and induced adipocyte-specific genes. The direct reprogramming protocol of human dermal fibroblasts to adipocyte-like cells we established is simple, fast and efficient, and has the potential to generate cells which can serve as a tool to address some, though not all, aspects of adipocyte function in the presence of endogenous disease-causing mutations.

## Abstract (precis)

**Summary:** Human dermal fibroblasts can be reprogrammed efficiently to adipocyte-like cells that have utility for modelling some aspects of human monogenic metabolic disease.

## INTRODUCTION

Adipose tissue is a crucial nexus in the regulation of intermediary metabolism, orchestrating metabolic adaptation to widely fluctuating nutritional demands. It serves not just as an insulin-regulated energy buffer, storing energy-dense triglyceride in times of plenty, and releasing it as free fatty acids and glycerol in times of privation, but also engages in more complex crosstalk with metabolically important organs such as liver, muscle and brain. It achieves this through fluxes of other metabolites and of a wide variety of established or putative ‘adipokine’ hormones, pre-eminent among which are leptin and adiponectin (Rosen and Spiegelman, 2014; Fasshauer and Blüher, 2015; Cohen and Spiegelman, 2016). In keeping with this key role of adipose tissue in metabolic homeostasis, anatomical deficiency or dysfunction of adipose tissue is now known to be the primary abnormality in many single gene forms of dyslipidaemic insulin resistance, and a major contributor to insulin resistance in more complex pleiotropic syndromes (Garg, 2011; Robbins and Savage, 2015; Hussain and Garg, 2016). Growing population-wide genetic evidence has also implicated adipose dysfunction as the mediator of the associations observed among a raft of common genetic variants and insulin resistance (Yaghootkar et al., 2014, 2016; Shungin et al., 2015; Kilpeläinen et al., 2016; Lotta et al., 2017). These findings have created a growing need for readily available and robust human adipocyte models in which the effects of natural genetic variation can be studied.

Several murine preadipocyte cell lines, most prominently the 3T3-L1 line (Green and Meuth, 1974), have been heavily studied, and have been the major tool in the delineation of the transcriptional network underlying adipogenesis over the past three decades (Rosen and MacDougald, 2006; Poulos et al., 2010). Some human cell lines also exist, including hMADS cells, isolated from the stromovascular fraction of infant adipose tissue (Rodriguez et al., 2004; Bezaire et al., 2009), SGBS cells, cultured from an individual with an overgrowth syndrome of undefined molecular aetiology (Fischer-Posovszky et al., 2008), and ChubS7 cells, primary preadipocytes immortalised with telomerase and HPV-E7 expression (Darimont et al., 2003); however, most are either too fragile to permit genetic engineering and clonal selection while retaining differentiation potential, or else recapitulate only poorly key behaviours of bona fide adipocytes, including secretion of leptin, insulin-stimulated glucose uptake and suppression of lipolysis. Primary cells can be used, but establishing cultures involves invasive biopsy procedures, which are impractical or hazardous in those with lipodystrophy, and the resulting cells can only be studied for relatively short periods before replicative senescence and differentiation failure supervene.

A further option is to utilise stem cells, whether embryonic stem cells (the use of which is practically constrained by licensing and ethical considerations) or induced pluripotent stem cells (iPSCs). Protocols for differentiating human stem cells to adipocyte-like cells have been published (Ahfeldt et al., 2012; Noguchi et al., 2013; Lee and Cowan, 2014; Mori et al., 2016; Guénantin et al., 2017); however, although this approach can generate cells with close transcriptional and functional similarities to primary adipocytes (Ahfeldt et al., 2012), it is time consuming and relatively labour intensive, taking several months to achieve, whether via reprogramming of primary human cells or via CRISPR/Cas9-mediated editing of an established iPSC line (Ahfeldt et al., 2012). In addition, generation of disease-specific iPSCs is often complicated by factors such as low efficiency of reprogramming, variability in protocols and the high cost of cell culture. Moreover, cellular changes during the reprogramming process, culture-induced spontaneous differentiation and differences in genetic background can constrain their use in cellular phenotyping of disease (Soldner and Jaenisch, 2012). These disadvantages are often prohibitive, rendering screening of numerous individuals laborious and impractical.

A faster approach to utilising primary human cells is direct reprogramming to the differentiated cell type of interest, thereby bypassing the generation of true pluripotent cells as intermediates (Vierbuchen and Wernig, 2011; Tanabe et al., 2015). This approach generally exploits knowledge about transcriptional and hormonal regulation of differentiation, and potentially offers the advantages of fast dynamics and high efficiency, making relatively rapid screening of numerous individuals feasible (Vierbuchen and Wernig, 2011). The detailed knowledge of the stereotyped transcriptional network driving adipogenesis that has accrued over the past 25 years, and particularly the dominant role of peroxisome proliferator-activated receptor gamma (PPARγ) as the master adipogenic transcription factor (Rosen and MacDougald, 2006), means that direct reprogramming of suitable cells to adipocytes is particularly tractable. Moreover, scattered evidence suggests that fibroblasts cultured from human dermis, obtainable by a quick and simply biopsy procedure, include mesenchymal progenitor cells (Feisst et al., 2014). We thus set out to assess whether readily cultured human dermal fibroblasts could be driven directly to become adipocytes as a cheaper and more convenient way of studying human disease variants in a disease-relevant cell type.

## RESULTS

### Direct reprogramming of dermal fibroblasts into adipocyte-like cells

**Fig. 1.**
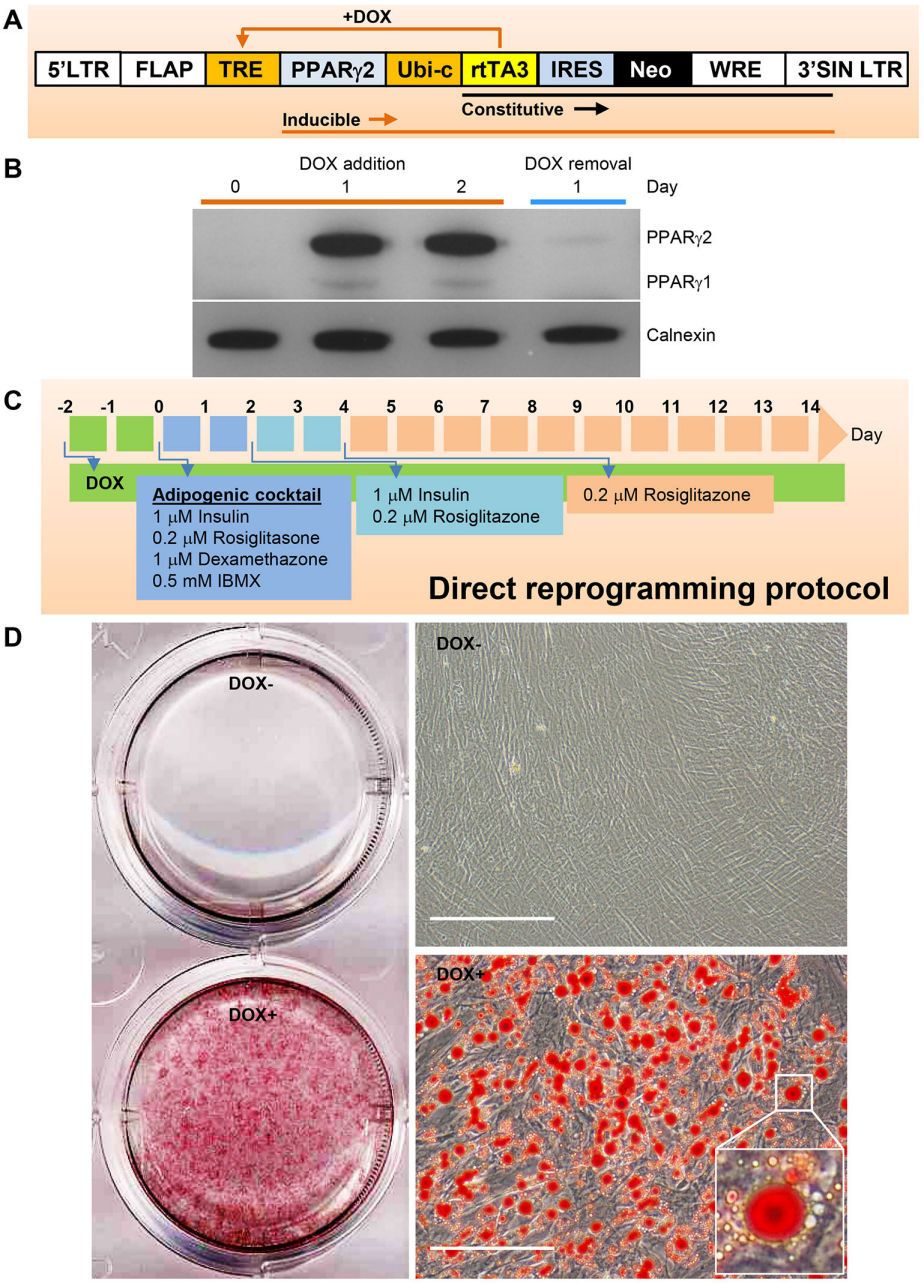
**Direct reprogramming of human dermal fibroblasts into adipocyte-like cells using inducible lentiviral PPARγ2 overexpression.** (A) Schematic showing predicted constitutive (black) and doxycycline (DOX)-induced (orange) transcripts from the pSLIK lentivirus. (B) Western blot analysis of kinetics of PPARγ2 overexpression in human dermal fibroblasts transduced with pSLIK-PPARγ2 recombinant lentivirus, which were cultured in the presence of DOX (1 µg/ml) followed by DOX withdrawal for the indicated length of time. Equal loading was revealed by anti-calnexin antibody. (C) Schematic showing the direct reprogramming protocol, which consists of DOX induction for 2 days, followed by 2 days culture in the presence of adipogenic cocktail and 2 days in the presence of insulin and rosiglitazone, and then rosiglitazone only for the rest of the culture. (D) Oil Red O staining showing the successful direct conversion of human dermal fibroblasts into triglyceride-laden adipocyte-like cells. Scale bars: 200 µm. The high magnification inset demonstrates a representative adipocyte with a large dominant lipid droplet.

We first constructed a recombinant lentivirus (pSLIK-PPARγ2) incorporating the longer PPARγ2 splice variant of the *PPARG* gene, which is highly expressed in adipose tissue (Tontonoz and Spiegelman, 2008) (Fig. 1A). This vector permitted conditional overexpression of PPARγ2 under the control of doxycycline via a third-generation version of the reverse Tet transactivator (rtTA3), which has been shown to have improved doxycycline sensitivity and activity (Das et al., 2004; Markusic et al., 2005; Shin et al., 2006). Western blot analysis showed that PPARγ2 overexpression in pSLIK-PPARγ2-transduced cells was induced 1 day after addition of doxycycline (1 µg/ml) and was maintained strongly in the presence of doxycycline (Fig. 1B). PPARγ1 was also detected, although at a much lower expression level (Fig. 1B); however, it was not possible to discriminate whether this resulted from minor usage of a second *PPARG* translational start codon in the transduced cDNA, or upregulation of endogenous PPARγ1 by PPARγ2 overexpression. Both PPARγ2 and low level PPARγ1 overexpression were rapidly turned off by removing doxycycline from the culture medium, becoming almost undetectable 1 day after doxycycline withdrawal (Fig. 1B).

To determine whether pSLIK-PPARγ2-transduced dermal fibroblasts can be directly reprogrammed into adipocyte-like cells, we subjected the stable cell lines to doxycycline induction for 2 days, followed by exposure to a standard adipogenic protocol. This consisted of use of an adipogenic cocktail [1 µM insulin, 200 nM rosiglitazone, 1 µM dexamethasone, 0.5 mM 3-isobutyl-1-methylxanthine (IBMX)] for 2 days followed by insulin and rosiglitazone at the same concentrations for 2 days, with rosiglitazone only for the rest of the culture period (Fig. 1C). Doxycycline was included throughout to maintain PPARγ2 overexpression. Morphological changes (loss of typical spindle-shaped, bipolar and refractile characteristics to become rounder and less refractile) were noticed as early as 1 day after adipogenic cocktail addition and were accentuated on day 2, when the appearance of small lipid droplets was noted. During the course of adipogenic differentiation, lipid droplets continued to accumulate and merge, with most lipid droplet-containing cells containing a dominant lipid droplet surrounded by many small droplets. Nearly homogenous differentiation and lipid accumulation were confirmed by Oil Red O staining (Fig. 1D). Stable cell lines remained undifferentiated in the absence of doxycycline, despite being subjected to the adipogenic protocol (Fig. 1D). We observed that the majority of reprogrammed cells which bear a prominent large lipid droplet were still alive at day 70, when cultures were terminated (Fig. S1).

**Fig. 2.**
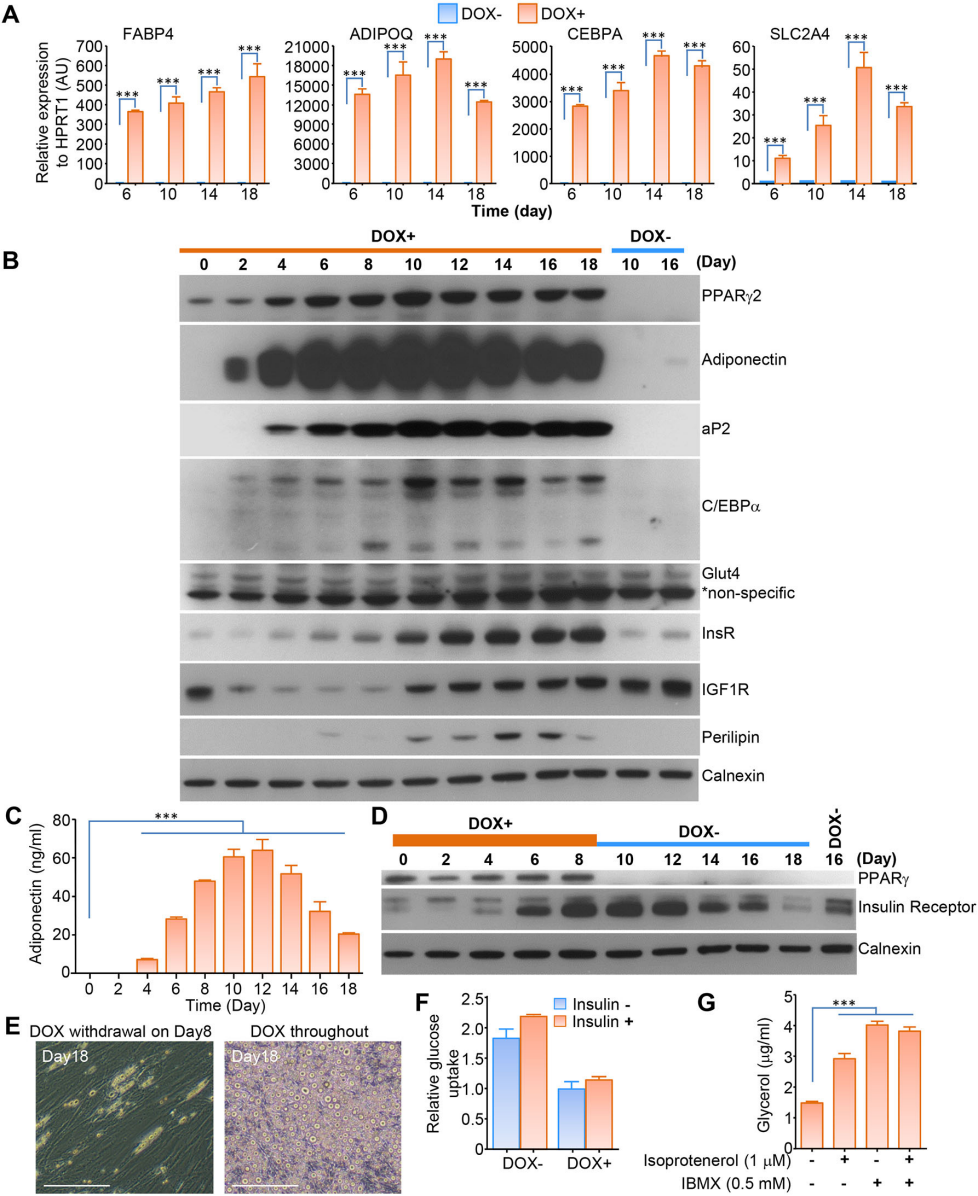
**Characterisation of direct reprogrammed adipocyte-like cells.** (A) Quantitative RT-PCR analysis of expression of marker genes of white adipocytes (*FABP4*, *ADIPOQ*, *CEBPA* and *SLC2A4*) at four different differentiation time points. (B) Western blot analysis of key proteins in adipocyte-like cells during direct reprogramming up to day 18, using antibodies as indicated. (C) Adiponectin secretion from direct reprogrammed adipocyte-like cells in 48-h culture medium was determined with DELFIA. (D) Downregulation of PPARγ2 and then *INSR* expression after removal of DOX from the culture medium. (E) Delipidation was also observed in reprogrammed adipocyte-like cells. Images were taken 10 days after DOX withdrawal (left) or with DOX included in the culture medium throughout (right). Scale bars: 100 µm. (F) Glucose uptake assay. (G) Lipolysis assay of direct reprogrammed adipocyte-like cells treated with isoprotenerol and/or IBMX. Data are mean±s.e.m. from three independent experiments (***P<0.001, Student's *t*-test).

Quantitative real-time PCR revealed that reprogrammed lipid droplet-containing cells expressed a panel of adipocyte marker genes, including *FABP4* (encoding aP2), *ADIPOQ* (encoding adiponectin), *CEBPA* (encoding C/EBPα) and *SLC2A4* (encoding GLUT4) (Fig. 2A); however, *LEP* expression (encoding leptin) was suppressed, even compared with the low baseline in skin cells (Fig. S2A). Expression of brown adipocyte marker genes was variable, with UCP1 strongly induced transcriptionally, but other genes showed either no increase (*DIO2* and *CYC1*) or only a moderate increase (*ELOVL3*) (Fig. S3A). Expression of aP2, adiponectin and C/EBPα was confirmed by western blot analysis (Fig. 2B); however, there was no evidence of UCP1 protein expression (Fig. S3B). Western blot analysis also revealed that insulin receptor and perilipin protein expression were induced in reprogrammed ‘adipocyte-like’ cells (Fig. 2B), while IGF1 receptor expression was markedly supressed up to day 8, after which time it gradually returned to a level of expression close to that of undifferentiated cells (Fig. 2B). GLUT4 protein expression showed no significant change during the course of exposure to adipogenic medium (Fig. 2B).

Secretion of adiponectin by reprogrammed adipocyte-like cells was determined by dissociation-enhanced lanthanide fluorescent immunoassay (DELFIA) in media exposed to cells for 48 h. Adiponectin became detectable on day 4 and increased up to day 12, after which time it decreased, in good agreement with western blot analysis results (Fig. 2C). High sensitivity DELFIA showed leptin, by contrast, to be suppressed from a low baseline level to be barely detectable in adipocyte-like cells (Fig. S2B).

It is believed that once the adipogenic programme is initiated, a cascade of pro-adipogenic factors is activated that eventually leads to self-sustaining mutual upregulation of PPARγ2 and C/EBPα (Rosen and MacDougald, 2006; Lefterova and Lazar, 2009). We thus sought to determine whether the adipocyte-like phenotype was maintained in the absence of exogenous PPARγ2 expression, as has been reported in stem cell-derived adipocytes (Ahfeldt et al., 2012). Withdrawal of doxycycline from adipocyte-like cells at 10 days of differentiation led to abrupt cessation of PPARγ2 expression, however, with attendant decreased expression of insulin receptor (Fig. 2D) and delipidation of adipocyte-like cells (Fig. 2E).

Insulin-stimulated glucose uptake into adipocytes accounts for only a small proportion of insulin-stimulated glucose disposal compared with skeletal muscle, the major site of insulin-stimulated, GLUT4-mediated glucose disposal (Klip and Paquet, 1990). Nevertheless, glucose uptake into adipose tissue is important for *de novo* lipogenesis. Furthermore, as murine 3T3-L1 adipocytes in culture show notably robust insulin-stimulated glucose uptake, unlike primary muscle cells or muscle-derived cell lines, it has been in adipocytes that many of the key studies of the biochemistry and cell biology of insulin-stimulated glucose uptake have been undertaken (Saltiel and Kahn, 2001). The degree of insulin stimulated glucose uptake in human adipocyte cell lines is generally much smaller than in 3T3-L1 adipocytes (commonly less than twofold compared with five- to tenfold). Despite strong upregulation of insulin receptor expression using the adipogenic protocol in conjunction with PPARγ2 overexpression in this study, we did not observe any insulin-stimulated glucose uptake in the reprogrammed adipocyte-like cells (Fig. 2F).

Farmer and co-workers (El-Jack et al., 1999; Hamm et al., 1999) previously reported that although PPARγ overexpression was sufficient to drive both NIH-3T3 and Swiss-3T3 cells to assume adipocyte-like morphology and express key adipocyte marker genes such as *Fabp4*, insulin-dependent glucose uptake was seen only in differentiated Swiss-3T3 cells. Lack of insulin-dependent glucose uptake in differentiated NIH-3T3 cells was shown to result from absence of C/EBPα and GLUT4 expression, as overexpression of C/EBPα was sufficient to induce GLUT4 expression and insulin-dependent glucose uptake in the resulting differentiated NIH-3T3 cells. We therefore assessed the effect of combining adipogenic induction of skin cells with overexpression of C/EBPα either alone or in combination with PPARγ2 (Fig. S4A). We observed that conversion into adipocyte-like cells by C/EBPα overexpression alone was inefficient, and that the resulting adipocyte-like cells generally accumulated small lipid droplets with no dominant droplet as seen in adipocyte-like cells by PPARγ2 overexpression (Fig. S4B). Moreover, immunoblotting revealed that neither C/EBPα alone nor C/EBPα with PPARγ2 (Fig. S4C) enhanced GLUT4 expression in the reprogrammed cells (Fig. S4D).

A further characteristic feature of mature white adipocytes is the ability to undergo lipolysis with release of glycerol upon stimulation of certain adrenergic receptors (predominantly β3 in mice and α2 in humans) (Wang et al., 2008). Lipolysis assays demonstrated that the reprogrammed adipocyte-like cells were capable of undergoing lipolysis when exposed either to the adrenergic agonist isoproterenol or the phosphodiesterase inhibitor IBMX. No synergy was observed between treatments (Fig. 2G).

### Comparison with stem cell-derived human adipocytes

Having demonstrated that adipocyte-like cells derived directly from dermal fibroblasts exhibit some but not all characteristics of adipocytes, we sought to compare them to adipocytes derived from human embryonic stem cells using a protocol closely similar to that previously described (Fig. S5A). Stem cells were first differentiated to mesenchymal stem cells before acute infection with the same conditional PPARγ2-expressing lentivirus as used in dermal fibroblasts. Adipogenic differentiation was then undertaken in the presence of doxycycline for 16 or 21 days. Like the dermal fibroblast-derived cells, the embryonic stem cell-derived cells accumulated neutral lipids (Fig. S5B), strongly induced a panel of adipocyte-specific genes at mRNA level (Fig. S6A), as well as adiponectin secretion (Fig. S6B), and exhibited isoproterenol-stimulatable lipolysis (Fig. S6C). Also, like dermal fibroblast-derived adipocyte-like cells, these cells did not upregulate leptin expression, and although they did exhibit significant upregulation of glucose uptake on insulin stimulation, the increase was modest, at only 1.7-fold (Fig. S6D). Contrary to previous findings, neither the expression of mature adipocyte genes nor the expression of endogenous PPARγ expression were robustly maintained after 1 week of doxycycline deprivation, with gene expression of all markers falling sharply in all cell lines tested (Fig. S7). Cellular lipid droplets were preserved after 1 week of doxycycline deprivation, however (Fig. S8), unlike in dermal fibroblast-derived adipocyte-like cells.

### Reprogramming of dermal fibroblasts derived from patients with genetic diseases

**Fig. 3.**
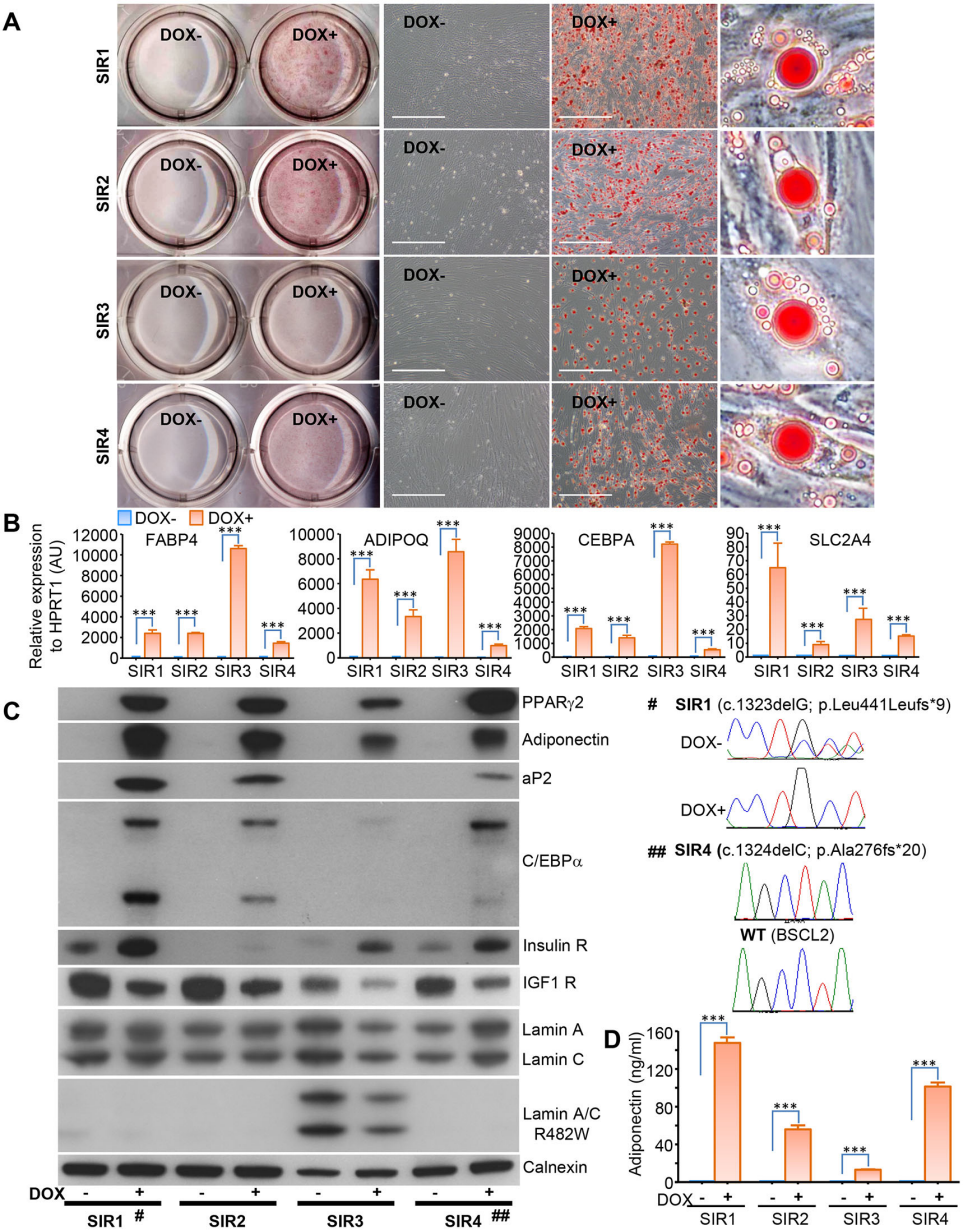
**Direct reprogramming of dermal fibroblasts derived from patients with metabolic diseases** **caused by** **mutations on PPARγ, insulin receptor, lamin and BSCL2.** (A) Oil Red O staining showing that the four patient cell lines (SIR1-SIR4) were all successfully converted into adipocyte-like cells using our direct reprogramming protocol, as schematised in Fig. 1. Scale bars: 200 µm. (B) Quantitative RT-PCR analysis of the expression of white adipocyte marker genes. (C) Western blot analysis of key proteins in undifferentiated control cells (DOX−) and reprogrammed adipocyte-like cells (DOX+). Sequencing confirmation of mutations on *PPARG* and *BSCL2* genes is shown for SIR1 and SIR4, respectively. (D) Adiponectin secretion from direct reprogrammed adipocyte-like cells in 48-h culture medium was determined with DELFIA. Data are mean±s.e.m. from three independent experiments (***P<0.001, Student's *t*-test).

We next sought to determine whether the direct reprogramming approach could be applied to human cells harbouring known mutations associated with Mendelian forms of severe insulin resistance, and whether disease-relevant phenotypic abnormalities could be observed in the resulting adipocyte-like cells. The genetic defects studied, and the clinical phenotypes of the donors, which included varying degrees of lipodystrophy in many cases, are summarised in Table S1. We found that cell lines harbouring pathogenic mutations in *PPARG*, *INSR*, *LMNA*, *BSCL2*, *PIK3R1*, *WRN*, *BLM*, *NSMCE2 ALMS1* or *PCYT1A* all exhibited lipid accumulation upon PPARγ2 overexpression and culture in adipogenic conditions (Fig. 3; Figs S9 and S10), despite many of these Mendelian disorders featuring some degree of failure of adipose tissue accumulation, ranging from generalised moderate paucity in the case of insulin receptoropathy, through partial lipodystrophy in the case of *PPARG* and *LMNA*, to severe generalised lipodystrophy in the case of *BSCL2*. Indeed, despite the severity of the lipodystrophy associated with biallelic *BSCL2* mutations, lipid droplets were indistinguishable in size and morphology from those in wild-type cells treated the same way (compare Fig. 3A and Fig. 1D). Adipocyte marker gene and protein expression (*FABP4*, *ADIPOQ* and *CEBPA*) were also strongly induced in all cells assessed (Fig. 3B,C), with the exception of *FABP4* in LMNA mutant cells. Adiponectin secretion was also detected by DELFIA in culture media (Fig. 3D). Induction of insulin receptor and suppression of IGF1 receptor expression, as seen before, were observed in patient cell lines, with the exception of the line harbouring biallelic INSR mutations, in keeping with the known effects of these INSR mutations to reduce protein expression of the receptor (Fig. 3C). Expression of the pathogenic p.Arg482Trp LMNA variant was shown in the relevant cell line using a mutation-specific antibody (Fig. 3C), while cDNA sequencing confirmed expression of the *PPARG* and *BSCL2* variants studied (Fig. 3E). Notably, however, expression of the *PPARG* mutant was no longer detectable after doxycycline addition, consistent with a high level of overexpression of wild-type protein. Reprogrammed fibroblasts from a patient with Alström syndrome and biallelic *ALMS1* mutations also displayed absent ALMS1 immunostaining at the centrosome (Fig. S9).

## DISCUSSION

Study of monogenic human disorders has unequivocally established impaired adipose tissue development or adipose dysfunction to be sufficient to produce severely insulin resistant diabetes, dyslipidaemia, fatty liver and ovulatory dysfunction (Semple et al., 2011). Emerging evidence suggests moreover that a subset of common human genetic variants confer genetic susceptibility to prevalent metabolic syndrome through a similar ‘lipodystrophic’ mechanism (Yaghootkar et al., 2014, 2016; Shungin et al., 2015; Kilpeläinen et al., 2016; Lotta et al., 2017). To pursue this notion mechanistically, and to test a growing repertoire of candidate ‘lipodystrophy’ susceptibility genes, requires study of gene variants in a relevant adipocyte or preadipocyte context, ideally with preservation of gene dosage and genomic context, and in a system that is scalable, to permit interrogation of multiple variants.

Primary preadipocytes, or adipose-derived stromal vascular cells, are obtained by invasive tissue biopsy, show variable but often low differentiation efficiency and can only be studied for a short period in culture. Mesenchymal stromal cells from bone marrow or other sources can also be differentiated into adipocytes, but similarly have limited proliferative and differentiation capacity (Mauney et al., 2007). Early attempts to generate adipocytes by differentiation of pluripotent human embryonic stem cells and iPSCs were also inefficient (Taura et al., 2009), but this approach was refined more recently by using conditional overexpression of PPARγ2 in conjunction with chemical induction of differentiation of iPSC-derived mesenchymal progenitor cells, yielding ∼90% of cells with white adipocyte characteristics (Ahfeldt et al., 2012). These cells were reported to show stable adipocyte-like characteristics even after turning off PPARγ2 transgene expression, and, moreover, exhibited insulin-stimulated glucose uptake and inducible lipolysis among other characteristics of mature adipocytes. Other reports have shown subsequently that adipocytes derived from iPSCs or embryonic stem cells can maintain adipocyte characteristics for 4 weeks after transplantation into mice (Noguchi et al., 2013), with one protocol reporting development *in vivo* of well vascularised beige-like adipose tissue, capable of beta-adrenergic-responsive glucose uptake (Guénantin et al., 2017). Nevertheless, these protocols are labour- and cost-intensive, prohibiting ready scaling for study of a wide range of mutations.

Direct reprogramming of somatic cells into other differentiated lineages has been achieved without an intermediate pluripotent stage for several cell types, in general relying on overexpression of key transcription factors previously established to be pivotal for development of the destination cells in question. Indeed, conversion of human dermal fibroblasts into haematopoietic progenitors (Szabo et al., 2010), various neurones (Caiazzo et al., 2011), cardiomyocytes (Nam et al., 2013), osteoblasts (Yamamoto et al., 2015), endothelial progenitor cells (Van Pham et al., 2016) and Schwann Cells (Sowa et al., 2017) has previously been described. The power of PPARγ2 as the ‘master regulator’ of adipogenic differentiation (Tontonoz and Spiegelman, 2008; Lefterova et al., 2014) makes this approach potentially appealing for adipocytes also, as illustrated by early studies in which its overexpression was used to turn mouse embryonic fibroblasts into adipocytes (Tontonoz et al., 1994).

We demonstrate that conditional expression of PPARγ2 in lentivirally transduced cells combined with a conventional adipogenic induction cocktail swiftly induced accumulation of large lipid droplets, as well as inducing expression of a panel of white, but not brown, adipocyte-specific genes, and high levels of adiponectin secretion. Another change in gene expression relevant to metabolic disease modelling was a marked increase in insulin receptor protein expression with concomitant downregulation of IGF1 receptor expression. Reprogrammed cells also exhibited isoproterenol or IBMX-stimulated lipolysis, in keeping with functional properties of mature white adipocytes.

Despite recapitulating some key aspects of adipocyte morphology, gene expression and function, however, the reprogrammed cells failed to recapitulate some important adipocyte behaviours relevant to at least some disease modelling. Thus, no insulin-stimulated increase in glucose uptake was seen in the adipocyte-like cells despite high levels of insulin receptor expression. Consistent with this, induction of GLUT4 protein expression was weak whether or not C/EBPα, which was previously suggested to induce GLUT4 expression (Hamm et al., 1999), was also overexpressed. The reprogrammed cells in this study also failed to induce leptin expression or secretion compared with nonreprogrammed dermal fibroblasts. Although PPARγ and CEBPα have been said to form a self-sustaining expression positive-feedback loop, we moreover found that the adipocyte-like phenotype of ‘reprogrammed’ fibroblasts was reversible when doxycycline-driven PPARγ2 expression was turned off, with rapid delipidation and loss of adipocyte gene expression, including endogenous *PPARG*, and of adiponectin secretion seen over the course of 1 week. For all the central importance of *PPARG* in the transcriptional control of adipogenesis, normal adipogenesis involves a complex cascade of activation of transcriptional networks as well as extensive chromatin remodelling (Siersbæk et al., 2012; Lefterova et al., 2014), and it is presumably the lack of such wider remodelling that determines the dependence of the adipocyte-like phenotype we report on continued artificial overexpression of *PPARG*. It will be of great interest to extend development of the model we describe to explore whether co-expression of other adipogenic factors can further enhance its robustness and fidelity in replicating mature adipocyte properties. One transcription factor of particular interest is *ZFP423*, which several studies have strongly implicated in priming perivascular cells for adipogenic differentiation (Gupta et al., 2012).

Collectively these findings raise the question as to whether the cells generated by the protocol described are truly ‘adipocytes’ or rather are simply cells with forced overexpression of *PPARG*-responsive genes, including those required for development of lipid droplets and adiponectin, which are known to be upregulated, and suppression of genes such as *LEP*, encoding leptin, which is known to be regulated negatively by PPARG (Hammarstedt et al., 2005). The latter possibility would still lend the cells utility for the study of certain aspects of adipocyte function, and indeed although the cells have shortcomings as bona fide adipocyte models, some of these are shared by other existing human adipocyte models. Although iPSC-derived cells have been reported to show insulin-stimulated glucose uptake (Ahfeldt et al., 2012), and although we replicated this finding, the degree of stimulation, as for other human adipocyte cell lines, was very small, such that the dynamic range of the response is unlikely to be of great use in assessing perturbations that induce insulin resistance. Furthermore, while leptin was reported to be expressed in iPSC-derived adipocytes, levels of protein secretion were extremely low (Ahfeldt et al., 2012). Finally, in our hands, even iPSC-derived adipocytes, although derived using a protocol very closely mimicking the prior study, showed marked loss of adipocyte gene expression, including endogenous PPARγ, after withdrawal of doxycycline-driven PPARG2 expression. These findings suggest that the advantages of using an iPSC-based approach to study human mutations in the correct genomic context are marginal only, at least when limited to studies in cell culture, and are substantially offset by the prolonged and expensive nature of the protocol.

We applied our reprogramming protocol to a panel of cells derived from patients with monogenic insulin resistance either attributable to an insulin signalling defect (*INSR*, *PIK3R1*), to primary lipodystrophy (*BSCL2*, *PCYT1A*, *PPARG* or *LMNA*), or forming part of a more complex, pleiotropic syndrome (*ALMS1*, *WRN*, *BLM*, *NSMCE2*). All cells could be transformed into lipid-laden, adipocyte-like cells, and while some variation in the efficiency of this process was observed across the panel of cells studied, similar variation was seen in wild-type cells, and is thus most likely attributable to intrinsic differences in cell proliferation and differentiation potential related to donor age or other factors not specific to the mutation harboured. The pathogenic genetic perturbation could be detected in four of the five cell lines in which it was assessed, whether this was loss of protein expression (compound heterozygous *INSR* or *ALMS1* mutations), expression of mutated protein (LMNA p.Arg482Trp heterozygosity) or expression of mutant mRNA (*BSCL2*). The only exception was cells with a pathogenic *PPARG* mutation, which could be detected in cDNA at baseline but not after *PPARG2* overexpression, which presumably swamped the endogenous mutant expression.

Surprisingly, no overt defects in lipid droplet accumulation was seen for any genotype, even where the corresponding human disorder features near complete absence of adipose tissue, as in generalised lipodystrophy caused by *BSCL2* mutations. The underlying mechanisms remain the subject of research, but several studies have invoked a crucial role for *BSCL2* in lipid droplet formation or function (Payne et al., 2008; Robbins and Savage, 2015). Although an obligate role for *BSCL2* in lipid droplet biogenesis would appear at odds with the observations in this study, lipid droplets can occur in some cellular contexts even in *BSCL2*-related lipodystrophy, such as in severely fatty liver cells. A previous study of adipogenesis of iPSC-derived human cells from a patient with BSCL2 mutations did show impaired adipose differentiation (Mori et al., 2016), while other studies have suggested that the defect in adipose differentiation of cells with knockout or knockdown of *BSCL2/Bscl2* can be partially overcome to some extent by activation of PPARG (Chen et al., 2009; Prieur et al., 2013). Our findings confirm that the *BSCL2* gene is dispensable for lipid droplet formation in the context of forced *PPARG* overexpression and application of an adipogenic protocol, but caution that some cell autonomous defects relevant to the *in vivo* human phenotype can be masked by the strength of *PPARG* activation employed in this model. For most of the other cell lines studied, the associated adipose phenotype is more complex, variable or age dependent, and calibration or adaptation of the reprogramming protocol might further enhance the value of the model in probing cell autonomous disease mechanisms.

In conclusion, we describe a protocol involving conditional PPARγ2 overexpression and a chemical adipogenic cocktail to turn dermal fibroblasts into lipid-laden adipocyte-like cells. These adipocyte-like cells expressed a panel of adipocyte-specific genes, showed upregulation of insulin receptor and downregulation of IGF1 receptor, secreted large amounts of adiponectin and showed robust lipolysis in response to adrenergic stimulation. On the other hand, we could not demonstrate insulin-stimulated glucose uptake nor meaningful levels of leptin expression of secretion. The protocol was successful when applied to a panel of cells harbouring monogenic defects causing severe insulin resistance, some with severe lipodystrophy, in humans. This simple protocol adds to the repertoire of cell models available to interrogate the consequences of genes involved in human metabolic disease in the appropriate genomic context and with endogenous expression levels. The approach might be most suited to examination of disorders of lipid droplet formation and function, of proximal insulin signalling, or, for example, to assess naturally occurring *ADIPOQ* variants for their impact on protein expression.

## MATERIALS AND METHODS

### Maintenance of dermal fibroblasts

All patient cell line studies were approved by the United Kingdom National Health Service Research Ethics Committee. The probands and families provided written informed consent, and studies were conducted in accordance with the principles of the Declaration of Helsinki. Dermal fibroblasts were cultured in Dulbecco's modified Eagle medium (DMEM; D6546, Sigma-Aldrich) supplemented with 10% foetal bovine serum (HyClone), 2 mM L-glutamine (G7513, Sigma-Aldrich) and 1% penicillin-streptomycin (P0781, Sigma-Aldrich) in a humidified incubator (37°C, 5% CO_2_). Cells were passaged once a week at a 1:4 split ratio.

### Inducible lenti-PPARγ2 production and generation of stable cell lines

The coding region of human PPARγ2 was amplified by PCR from a plasmid containing PPARγ open reading frame (a gift from Erik Schoenmakers, University of Cambridge) and cloned into pCR4TOPO vector (Zero Blunt TOPO PCR Cloning Kit, Invitrogen). After sequencing confirmation, the coding sequence was excised and inserted into the entry vector pEN-Tmcs (ATCC MBA-251, LGC Standards) using *Spe*I and *Not*I restriction sites. The expression cassette was then transferred to the pSLIK-Neo expression lentivector (ATCC MBA-236, LGC Standards) using site-specific recombination (Gateway LR Clonase II Enzyme Mix, Invitrogen). All clones were confirmed by restriction digest screening and sequencing. To produce recombinant lentivirus, 10 µg of pSLIK-PPARG plasmid together with 7.5 µg of each of the two packaging plasmids, pMDLg/pRRE and pRSVREV, and 5 µg of the plasmid coding for VSV-G envelope as well as 1 µg of pEGFP, were transfected into HEK293T cells on 10-cm culture dishes using CalPhos Mammalian Transfection Kit (Clontech). The transfection efficiency was monitored with GFP expression, revealed by fluorescent microscopy. The medium was replaced after 12 h with BioWhittaker Ultraculture Medium (Lonza) and 48 h post-transfection the viral supernatants were harvested, filtered through a 0.45 µm filter and concentrated by using Centricon Plus-70 Ultracel PL-100 (Millipore).

Dermal fibroblasts were trypsinised, seeded at ∼40% confluency and, after adhering onto the culture plates (∼4 h after seeding), transduced with the above concentrated lentiviral supernatant at low multiplicity of infection in the presence of 8 µg/ml polybrene. Six-well plates were transduced with 8 µl lenti-PPARγ virus per well in 0.8 ml culture medium and, after adding 1.2 ml fresh medium per well 6-8 h later, cells were cultured for 2 days. After removing the virus-containing medium, cells were then subjected to G418 (Sigma-Aldrich) selection at a final concentration of 800 µg/ml for 10 days. Stable cell lines were maintained in culture medium containing 200 µg/ml G418. Inducibility of PPARγ overexpression with doxycycline was assessed using western blotting.

### Inducible lenti-C/EBPα production and generation of stable cell lines

Human C/EBPα cDNA clone (TrueORF) was purchased from OriGene Technologies (SC303472, Rockville, USA). The cDNA was released from pCMV6-XL5 vector with *Eco*RI and *Xba*I restriction digestion, gel purified and then inserted into pEN-Tmcs vector that had been digested with the same restriction enzymes. After sequencing confirmation, the expression cassette was then transferred to the pSLIK-Hygro expression lentivector (ATCC MBA-236, LGC Standards) using site-specific recombination (Gateway LR Clonase II Enzyme Mix, Invitrogen). Lenti-C/EBPα production and generation of stable cell lines were carried out essentially in the same way as described above except that selection of stable cell lines was performed using hygromycin (200 mg/ml).

### Adipogenic differentiation of stable cell lines

Stable dermal fibroblasts were treated with doxycycline (1 µg/ml) for 2 days to induce PPARγ2 overexpression. Adipogenic differentiation was then initiated by addition of adipogenic cocktail (1 µM insulin, 200 nM rosiglitazone, 1 µM dexamethasone and 0.5 mM IBMX) for 2 days followed by insulin and rosiglitazone for 2 days and then rosiglitazone only for the rest of the culture. Doxycycline was included throughout the reprogramming process in order to maintain PPARγ2 overexpression in differentiating cells and the resulting mature adipocyte-like cells.

### Generation of stem cell-derived adipocytes

The protocol used to generate adipocytes from stem cells has been adapted from a previously published protocol (Ahfeldt et al., 2012). Briefly, embryoid bodies (EBs) were derived from H9 embryonic stem cells. Detached H9 colonies were then dissociated into clumps of 5-10 cells in EB formation medium consisting of 15% KnockOut serum replacement (Thermo Fisher Scientific) and 1% GlutaMAX (Thermo Fisher Scientific) in DMEM (Sigma-Aldrich) supplemented with 4 μM Y-27632 (Sigma-Aldrich). Cell clumps were then allowed to attach onto Ultralow attachment plates (Corning). The medium was refreshed after 24 h. The EB medium was replaced every other day for 5 days, after which the EBs were collected into a pellet, resuspended in EB plating medium consisting of 10% KnockOut Serum Replacement and 1% Glutamax in DMEM, and then plated onto 0.1% gelatin-coated plates. Fresh EB plating medium was added every other day until the EBs had attached and reached 90% confluency. Cells were then passaged using 0.125% trypsin-EDTA (Sigma-Aldrich) onto 0.1% gelatin-coated plates in adipocyte precursor (AP) medium containing 15% KnockOut Serum Replacement and 1× GlutaMAX in DMEM supplemented with 2.5 ng/ml bFGF (R&D Systems). The AP medium was refreshed every other day.

AP cells were then plated onto each well of a 0.1% gelatin-coated six-well plate in AP medium and transduced with lenti-PPARγ viruses in the presence of 8 µg/ml polybrene. To induce adipocyte differentiation, the AP medium was removed and adipocyte differentiation medium consisting of 15% KnockOut Serum Replacement (Lifetech), 0.5% non-essential amino acids (Invitrogen), 1% Glutamax (Lifetech) in DMEM (Sigma-Aldrich) supplemented with 1 μM dexamethasone (Sigma-Aldrich), 10 μg/ml insulin (Actrapid, Novo Nordisk), 0.5 μM rosiglitazone (Sigma-Aldrich) and 1 μg/ml doxycycline (Sigma-Aldrich) was applied to confluent transduced AP cells. The adipocyte differentiation medium supplemented with doxycycline was refreshed every other day for 16 or 21 days. Cells were then maintained for a further 7 days in adipocyte differentiation medium without doxycycline.

### Sequencing

Sequencing reactions were performed with primers specific to respective plasmids (Table S2) or PCR-amplified cDNA targets using BigDye terminator (Invitrogen) according to the manufacturer's protocol. PCR products were treated with shrimp alkaline phosphatase (EF0511, Fermentas) and ExoI (M0293L, BioLabs) to eliminate unincorporated primers and dNTPs prior to sequencing reaction. Sequencing extension products were purified using BigDye cleaning beads (BCB-100, MCLAB) and then analysed with an ABI3730 DNA analyser. DNA sequence data were analysed with Sequencher software (Gene Codes Corporation).

### mRNA expression analysis

Total RNA samples were prepared using RNeasy Mini Kits (Qiagen) with a DNase digestion step included to eliminate potential contaminating DNA and quantified spectrophotometrically on a NanoDrop ND-1000 (Thermo Fisher Scientific). First strand cDNA was reverse-transcribed from 400 ng total RNA using an ImProm-II Reverse Transcription System (Promega) with oligo(dT)_15_ as the primer, according to the manufacturer's protocol.

Quantitative RT-PCR was carried out using an ABI PRISM 7900 Sequence Detection System (Applied Biosytems) with a SYBR Green PCR Master Mix (Applied Biosystems) and gene-specific primers (Table S2). Primers were custom-designed and synthesised by Sigma-Aldrich. cDNA template (2 μl; diluted according to the relative expression level of each gene of interest) was used in a 12 μl total reaction volume in each well in a 96-well reaction plate. For every gene analysed in the present study, we performed dissociation curve analysis to ensure that the primers used did not form primer dimers. The transcripts of each gene were amplified in duplicate. Standard curves were constructed using serially diluted pooled cDNA samples. The relative expression levels were calculated against each gene's standard curve with the Ct values of each gene with the housekeeping gene *HPRT1* as a loading control. Expression of *HPRT1* had equal levels between control dermal fibroblasts and reprogrammed adipocyte-like cells

### Protein expression analysis

Cells were washed with ice-cold PBS and harvested in M-PER Mammalian Protein Extraction Reagent (Thermo Fisher Scientific), to which protease inhibitor mini complete cocktail (Roche) was added at a 1:7 ratio. Proteins were mixed with an equal volume of 2× SDS loading buffer and denatured at 100°C, and then resolved by SDS-PAGE and transferred to polyvinylidene fluoride membranes using an iBlot system (Invitrogen). Blots were blocked in TBST (50 mM Tris-HCl, pH 7.6, 150 mM NaCl, 0.1% Tween 20) containing 5% milk or BSA (Sigma-Aldrich) and probed overnight at 4°C with the following antibodies: anti-PPARγ (sc-7196, Santa Cruz Biotechnology, 1:500 dilution), anti-adiponectin (ab13881, Abcam, 1:1000 dilution), anti-Glut4 (ab654, Abcam, 1:4000 dilution), anti-C/EBPa (#2295, Cell Signaling Technology, 1:1000 dilution), anti-insulin receptor β (sc-711, Santa Cruz Biotechnology, 1:500 dilution), anti-IGF1 receptor (sc-713, Santa Cruz Biotechnology, 1:500 dilution), anti-calnexin (ab75801, Abcam, 1:10,000 dilution), anti-aP2 (sc-18661, Santa Cruz Biotechnology, 1:5000 dilution), anti-lamin A/C (sc-20681, Santa Cruz Biotechnology, 1:500 dilution), anti-perilipin (#3467s Cell Signaling Technology, 1:1000 dilution) and anti-lamin R482W (SAB4200422, Sigma-Aldrich, 1:5000 dilution). The bound primary antibodies were detected by horseradish peroxidase-conjugated secondary antibodies (1:5000 dilution), followed by Immobilon^TM^ Western chemiluminescent HRP substrate (WBKLS0500, Millipore).

### Glucose uptake assay

Adipocyte-like cells were serum starved in serum-free DMEM containing 0.5% BSA for 2 h with change after 1 h. Cells were then washed two times with HEPES glucose uptake buffer (120 mM NaCl, 5 mM KCl, 1.2 mM MgSO_4_, 10 mM NaHCO_3_, 1.3 mM CaCl_2_, 1.2 mM KH_2_PO_4_ and 20 mM HEPES, pH 7.8) containing 0.5% BSA, and then incubated in HEPES glucose uptake buffer containing 0.5% BSA in the absence or presence of 100 nM insulin for 30 min at 37°C. Glucose uptake was measured by incubating cells with 0.5 µCi/ml 2-deoxy-D-[^3^H]glucose (Perkin-Elmer) for 5 min at 37°C. After three washes with ice-cold PBS, cells were lysed with 0.5 M KOH for 30 min, and then neutralised with 0.5 M HCl before scintillation counting. Protein concentration in the cell lysate was determined using Coomassie Plus Reagent (Thermo Fisher Scientific).

### Lipolysis assay

Adipocyte-like cells were serum starved overnight (16 h) in serum-free DMEM containing 0.5% BSA. Cells were washed with KRB-HEPES buffer (118.5 mM NaCl, 4.75 mM KCl, 1.92 mM CaCl_2_, 1.19 mM KH2PO_4_, 1.19 mM MgSO_4_, 25 mM NaHCO_3_, 6 mM glucose and 10 mM HEPES, pH 7.4) containing 4% fatty-acid-free BSA, and then incubated in KRB-HEPES buffer with 4% fatty-acid-free BSA alone, with isoproterenol or with IBMX at 37°C, 5% CO_2_ for 3 h. The culture medium was collected for glycerol measurement using the free glycerol reagent (Sigma-Aldrich, F6428).

### Oil Red O staining

Cells were washed with PBS twice and fixed with 10% formalin solution (Sigma-Aldrich, neutral buffered) at room temperature for 10 min. After washing twice with PBS and twice with 60% isopropanol, cells were stained with Oil Red O working solution [six parts stock Oil Red O (0.25% in isopropanol): one part 60% isopropanol: three parts water] for 30 min. Stained cells were washed with 60% isopropanol and kept in PBS for scanning or microscopic imaging.

### Immunofluorescence analysis

Cells on coverslips were fixed in 4% paraformaldehyde in PBS for 10 min followed by one wash with TBS [50 mM Tris-HCl (pH 7.4), 150 mM NaCl], permeabilisation in 0.2% Triton X-100 in PBS for 5 min, three washes with TBS and quenching in fresh 0.1% sodium borohydride in TBS. Coverslips were blocked with blocking buffer (10% horse serum, 1% BSA, 0.02% NaN_3_, 1× PBS) for 1 h, washed with TBS and incubated with anti-ALMS1 (ab84892, Abcam, 1:1000 dilution) and anti-acetylated tubulin (T7451, Sigma-Aldrich, 1:1000 dilution) in 1% BSA in TBS overnight at 4°C. After washing, the cells were incubated with a 1:1000 dilution of Alexa Fluor 488-conjugated goat anti-mouse IgG (A11001, Invitrogen) and Alexa Fluor 555-conjugated goat anti-rabbit IgG (A21430, Invitrogen) for 45 min at room temperature in the dark, washed with TBS, mounted on glass slides using the ProLong Gold Antifade Reagent with DAPI (P36931, Invitrogen) and inspected with a Zeiss LSM510 Meta confocal laser scanning microscope.

### Statistical analysis

Data are expressed as mean±s.e.m. from at least three independent experiments (*n*≥3). Statistical analyses were performed in GraphPad Prism 5.0 (GraphPad Software, San Diego, CA, USA). Statistical significance was determined by pairwise comparisons using a two-tailed unpaired Student's *t*-test, with *P*<0.05 considered significant. The investigators were not blinded to the group allocation during the experiments.

## Supplementary Material

Supplementary information
