# Supplementary material for: Evaluation of human dermal fibroblasts directly reprogrammed to adipocyte-like cells as a metabolic disease model
Source: Dis Model Mech. 2017 Dec 1;10(12):1411–20. doi: 10.1242/dmm.030981 (PMC5769609; doi:10.1242/dmm.030981)
Supplement: Supplementary information [file dmm-10-030981-s1.pdf]

Supplementary Tables

Cell Line	Patient ID	Sex	Disease-related Genotype	Clinical Phenotype	Notes	Reference
SIR1	P894	M	*PPARG p.Leu441Leu_fs*9*<br>heterozygote	Familial partial lipodystrophy type 3		Unpublished
SIR2	P672	F	*INSR*<br>p.Val93Ala/<br>del exon 18	Rabson Mendenhall syndrome		P16 in Simpkin et al (Simpkin, Cochran et al. 2014)
SIR3	P0014	F	*LMNA*<br>p.Arg482Trp heterozygote	Familial partial lipodystrophy type 2	Common FPLD2 mutation	Kumar et al (Kumar, Durrington et al. 1996)
SIR4	P340	M	*BSCL2*<br>c.1324delC/ p.Ala276fs*20	Congenital generalised lipodystrophy	Most severe form of generalised lipodystrophy	unpublished
SIR5	P13	M	*ALMS1*<br>p.Thr3591Ilefs*5/p.Thr3591ilefs*5	Alström syndrome	Subject	P13 in (Chen, Geberhiwot et al. 2017)
SIR6	P621	F	*PIK3R1*<br>p.Tyr657* heterozygote	SHORT syndrome		Huang-Doran et al (Huang-Doran, Tomlinson et al. 2016)
SIR7		M	*WRN**	Werner syndrome		AG12795 (Coriell Institute)
SIR8		M	*BLM**<br>Homozygous for a 6 p deletion/ 7bp insertion at nucleotide 2,281of the open reading frame of the *RECQL3* gene	Bloom syndrome		GM02932 (Coriell Institute)
SIR9	P384	F	*NSMCE2*<br>*p.Ser116Leufs*18/ p.Ala234Glufs*4*	Primordial dwarfism with extreme IR		Patient 1 in Payne et al (Payne, Colnaghi et al. 2014)
SIR10	P689	F	*PCYT1A*<br>p.Glu280del<br>p.Ser333Leufs*164	Generalised lipodystrophy		Proband 2 in Payne et al (Payne, Lim et al. 2014)
SIR11	P616	F	*PCYT1A*<br>p.Glu280del/<br>p.Val142Met	Generalised lipodystrophy		Proband 1 in Payne et al (Payne, Lim et al. 2014)

Supplementary Table S1. Clinical and Genetic Diagnoses of Volunteers Contributing Dermal Fibroblasts for Study. *WRN and BLM deficient cells were purchased from Coriell Institute, catalogue numbers AG12795 and GM02932 respectively. Precise mutations were not available.

Disease Models & Mechanisms • Supplementary information

Gene	Forward primer (5′ to 3′)	Reverse primer (5′ to 3′)	Amplicon size (bp)
ADIPOQ	GGGTGGGCTCCTTACAGAAC	CTCTCTGTGCCTCTGGTTCC	89
FABP4	TACTGGGCCAGGAATTTGAC	TGCACATGTACCAGGACACC	91
CEBPA	GAGGAGGGGAGAATTCTTGG	GAGCGGTGAGTTTGCATTTC	92
GLUT4	AGTTCCAGCCATGAGCTACG	CCACGATGAACCAAGGAATG	95
LEP	CCAGGTAATGAGGGACTGGA	CATCCCTCACCTCCTTCAAA	88
UCP1	AGTGTGCCCAACTGTGCAAT	AATGACGTTCCAGGATCCAA	105
CYC1	AGAGCACGACCATCGAAAAC	TCAGGACTGACCACTTGTGC	104
ELOVL3	CCTGGGTCTTTCTTCTCAGC	GTGCTGTGGTGGTACCAGTG	103
DIO2	AGACCCAGCTCTGTTCCAAG	TCTGCTGTTGCTGCCTAAGA	119
HPRT1	AGTTCTGTGGCCATCTGCTT	TAGGAATGCAGCAACTGACA	96
BSCL2	ACGTGATCGGGTGCTGATGT	GGATGTTAACCTGCAAAGAG	538
PPARG	TCTCAGTGGAGACCGCCCAGGTT	GCAGTGGCTCAGGACTCTCT	280

Supplementary Table S2. Primers used for quantitative RT-PCR and mutation confirmation

Disease Models & Mechanisms • Supplementary information

Supplementary Figures

C005_Day70_DOX+

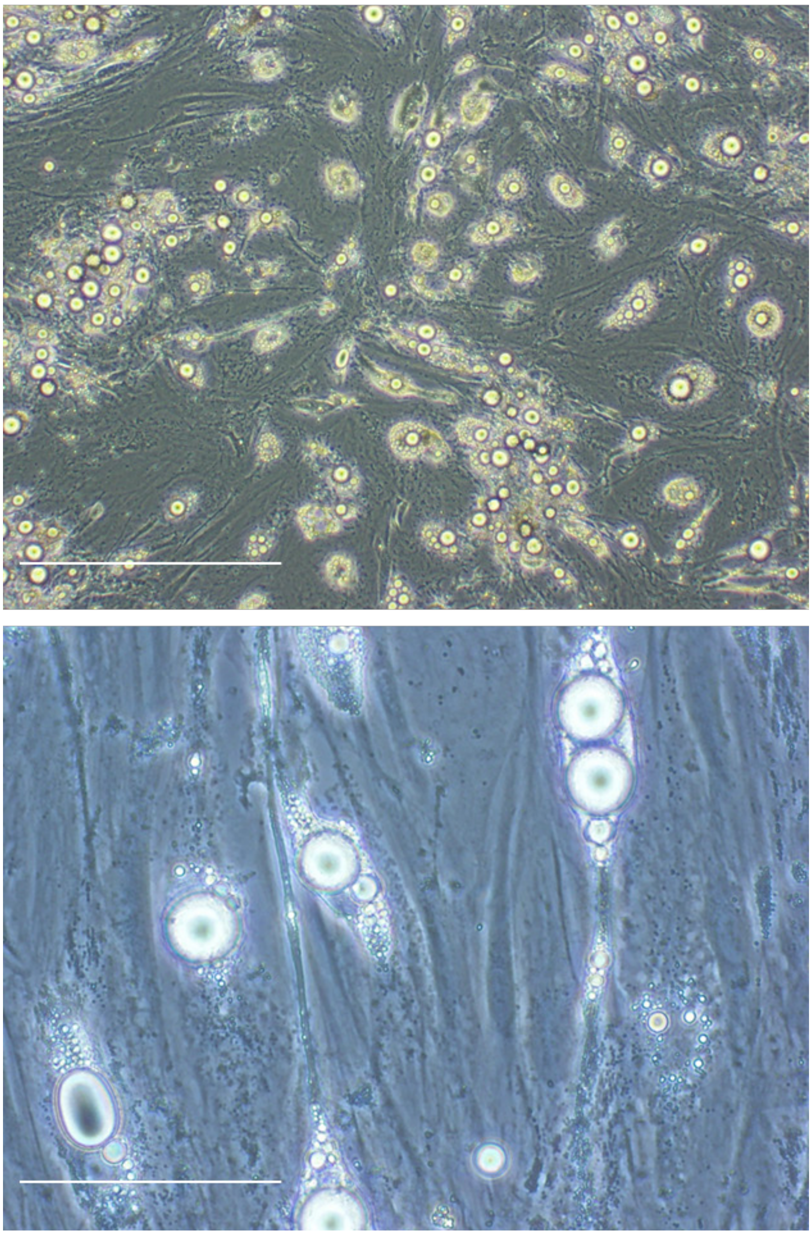

Supplementary Figure S1. Direct reprogrammed adipocyte-like cells can be maintained in culture for a long time in the continuous presence of doxycycline. Shown are two images at different magnifications for reprogrammed cells maintained in culture for 70 days. (scale bar: top, 200μm, bottom, 40μm).

Disease Models & Mechanisms • Supplementary information

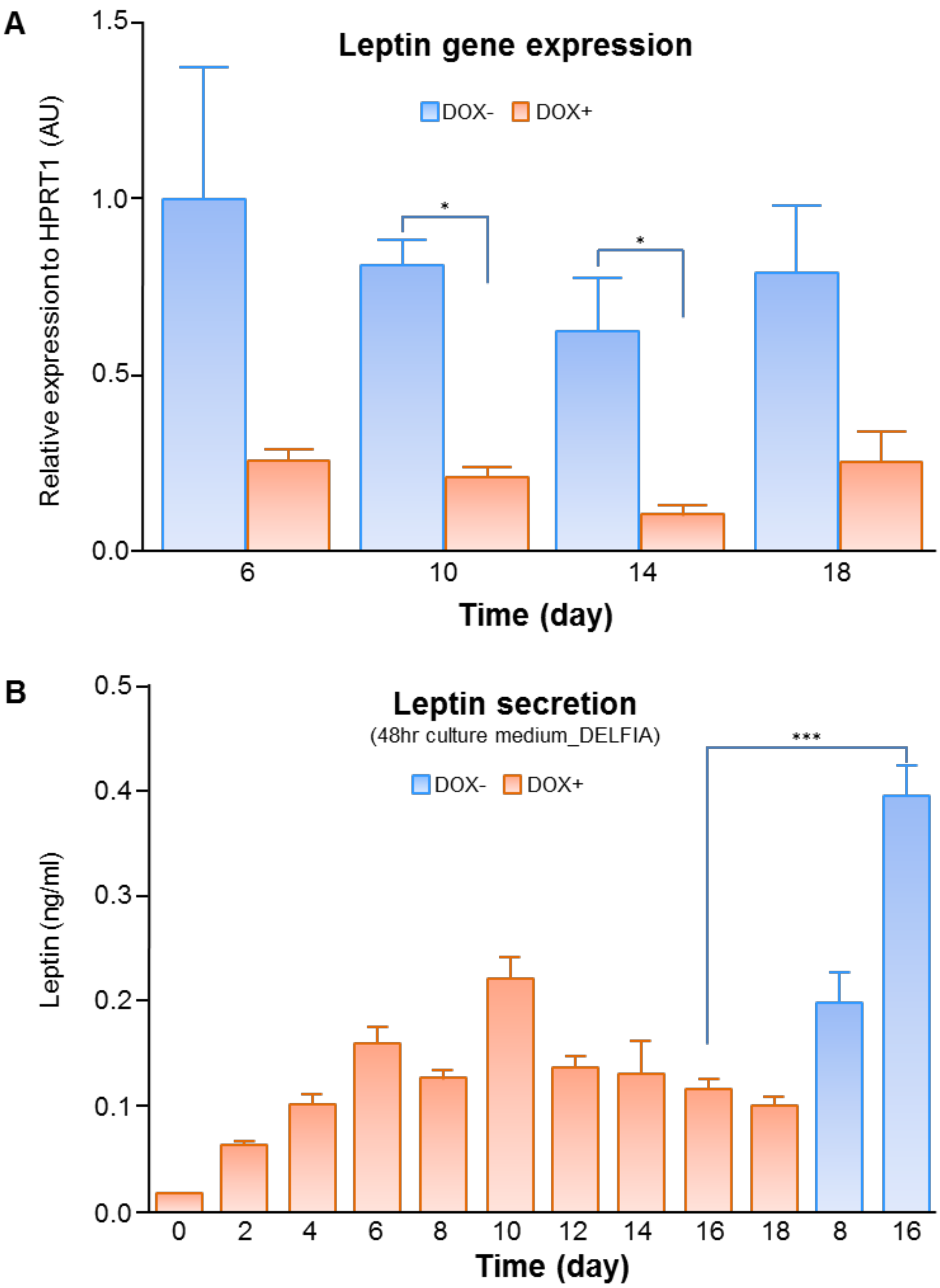

Supplementary Figure S2. Leptin gene expression of secretion of reprogrammed adipocyte-like cells. (A) Quantitative RT-PCR analysis of leptin gene expression. (B) Leptin secretion in 48 hour culture was determined with DELFIA. Data from three independent experiments are represented as mean ± SEM (*P<0.05, ***P<0.001, *t*-Test).

Disease Models & Mechanisms • Supplementary information

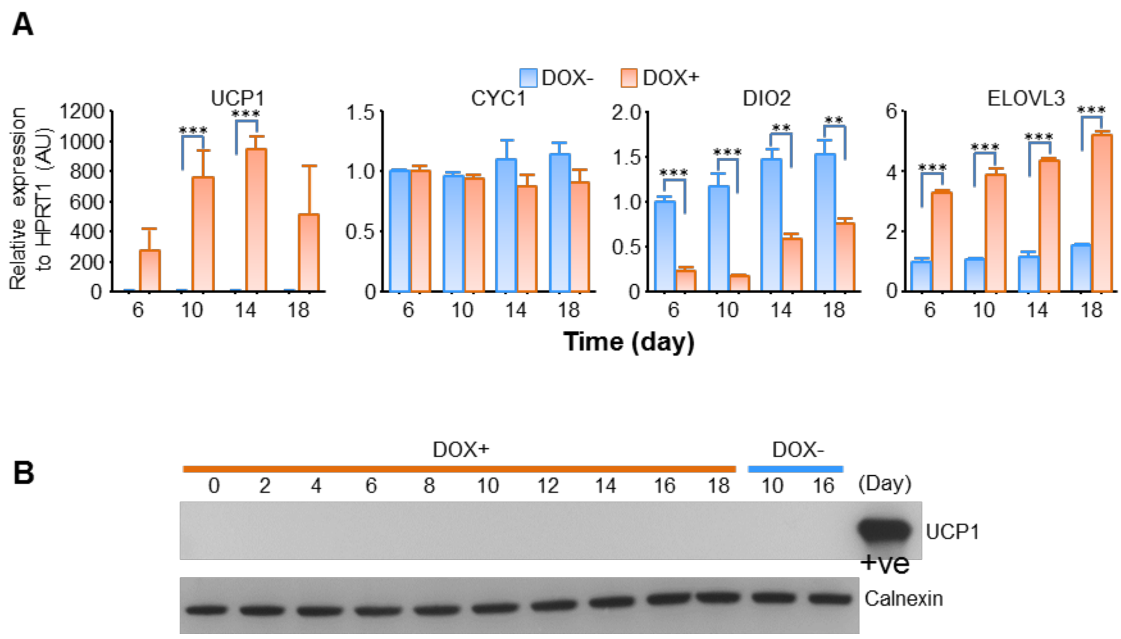

Supplementary Figure S3. Expression of brown adipocyte-specific genes (A) and UCP1 protein (B) in reprogrammed adipocyte-like cells. Expression was determined by quantitative RT-PCR and Western blotting respectively. A rat brown adipose sample was used as a positive control (“+ve”) in the Western blotting. Data from three independent experiments are represented as mean ± SEM (**P<0.01, ***P<0.001, *t*-Test).

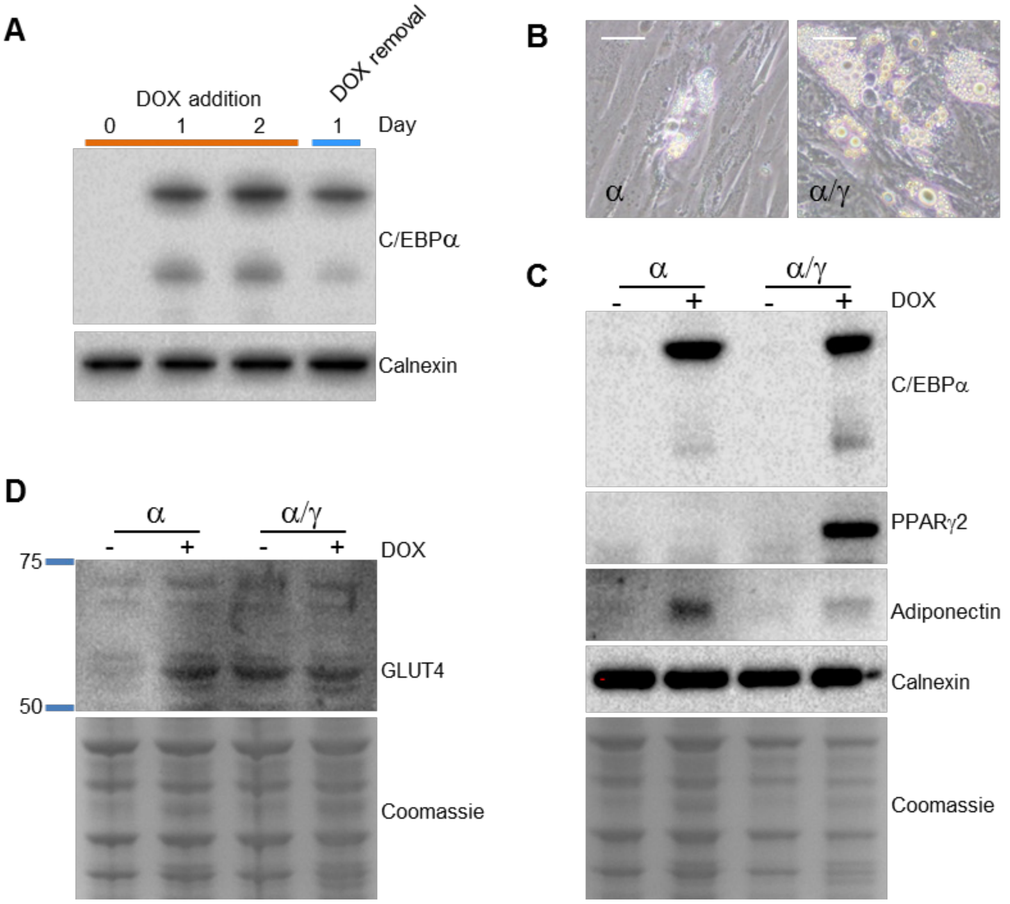

Supplementary Figure S4. Overexpression of C/EBPα and adipogenic differentiation of dermal fibroblasts infected with C/EBPα lentivirus alone or together with PPARγ2 lentivirus. (A) Western blot analysis of kinetics of PPARγ2 overexpression in human dermal fibroblasts transduced with pSLIK-C/EBPα recombinant lentivirus, which were cultured in the presence of DOX (1 μg/ml) followed by DOX withdrawal for the indicated length of time. Equal loading was revealed by calnexin antibody. (B) Representative images of adipocyte- like cells that accumulated lipid droplets upon DOX induction of C/EBPα overexpression (α) or co-overexpression of C/EBPα and PPARγ2 (α/γ) at adipogenic differentiation day 9. (scale bar: 10μm).

(C) Western blot analysis of C/EBPα, PPARγ2, and adiponectin in cells that had been subjected for adipogenic differentiation in the presence or absence of DOX for 9 days. Loading was revealed by calnexin antibody as well as Coomassie staining. (D) Western blot analysis of Glut4 expression in cells as in (C) above using unboiled samples. Equal loading was revealed by Coomassie staining. Note the uneven exposure of the blot that resulted in lighter bands coinciding with the first lane on the left hand side.

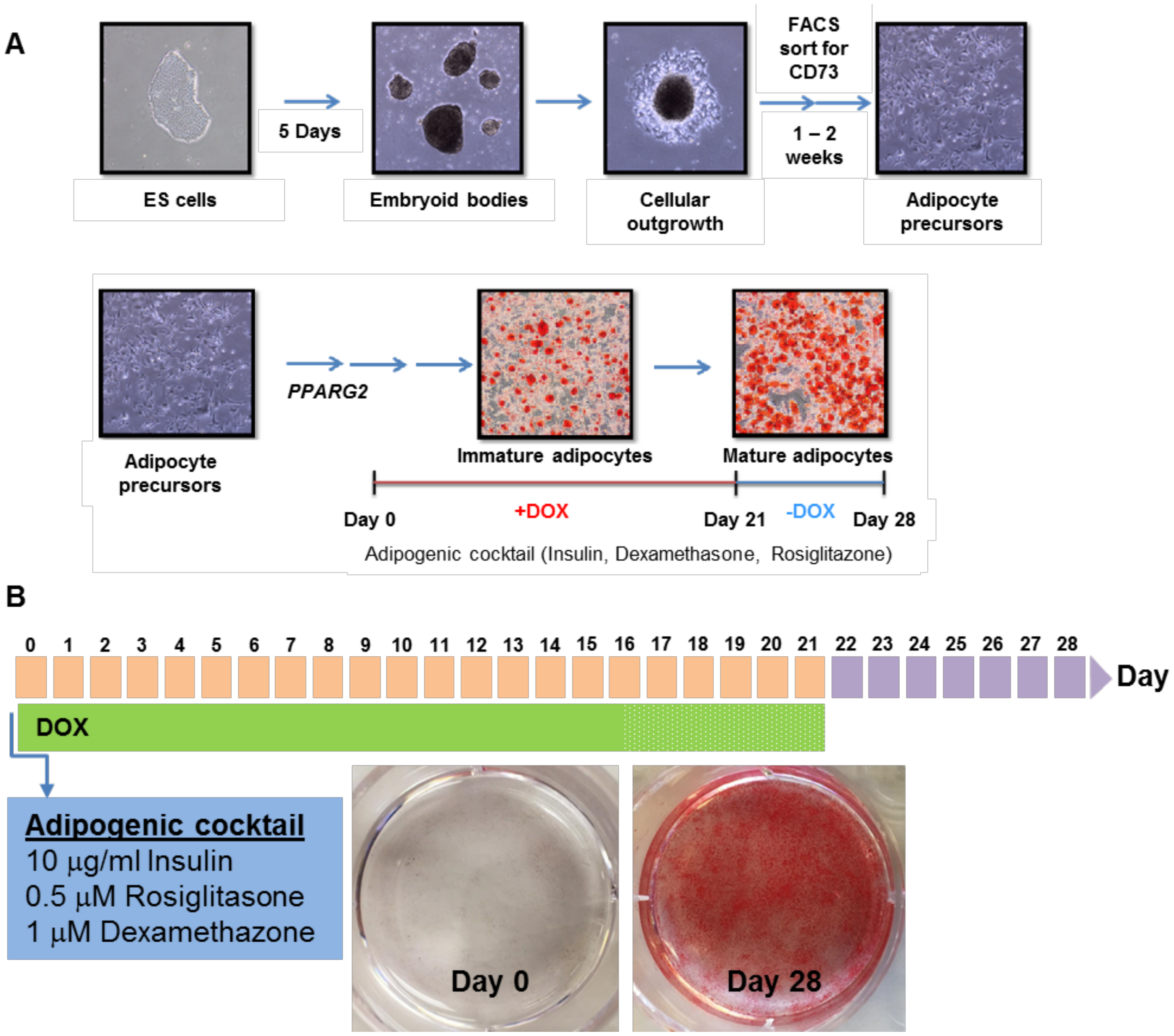

Supplementary Figure S5. Protocol of adipogenic differentiation via pluripotent stem cell stage. Overview of the protocol for deriving adipocytes from pluripotent stem cells. (A) Pluripotent stem cells were first differentiated into embryoid bodies (EBs) from which adipocyte precursor (AP) cells were derived. (B) Schematic showing adipogenic differentiation protocol which consists of DOX induction in the presence of adipogenic cocktail for the indicated time. DOX was included in the medium until day 16 or day 21 and cells were used for analysis either on the same day when DOX was withdrawn or 7 days after DOX withdrawal. Oil Red O staining showing a successful conversion of adipocyte precursors (Day 0) into triglyceride-laden adipocyte-like cells (Day 28).

Disease Models & Mechanisms • Supplementary information

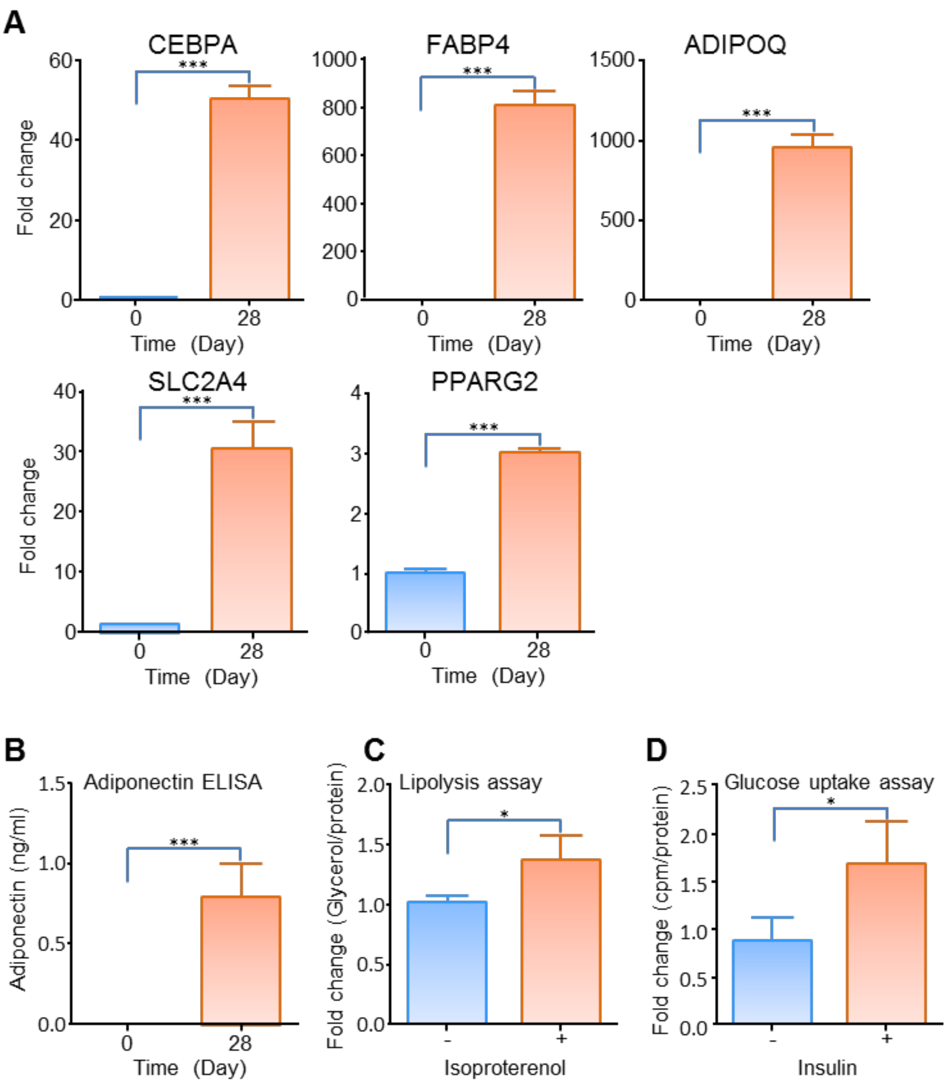

Supplementary Figure S6. Characterisation of adipocyte-like cells obtained using a protocol described in Supplementary figure 6. For details of quantitative RT-PCR (A), ELISA, lipolysis assay and glucose uptake assay (B), see above respective figure legends. Data from three independent experiments are represented as mean ± SEM (*P<0.05, ***P<0.001, *t*-Test).

Disease Models & Mechanisms • Supplementary information

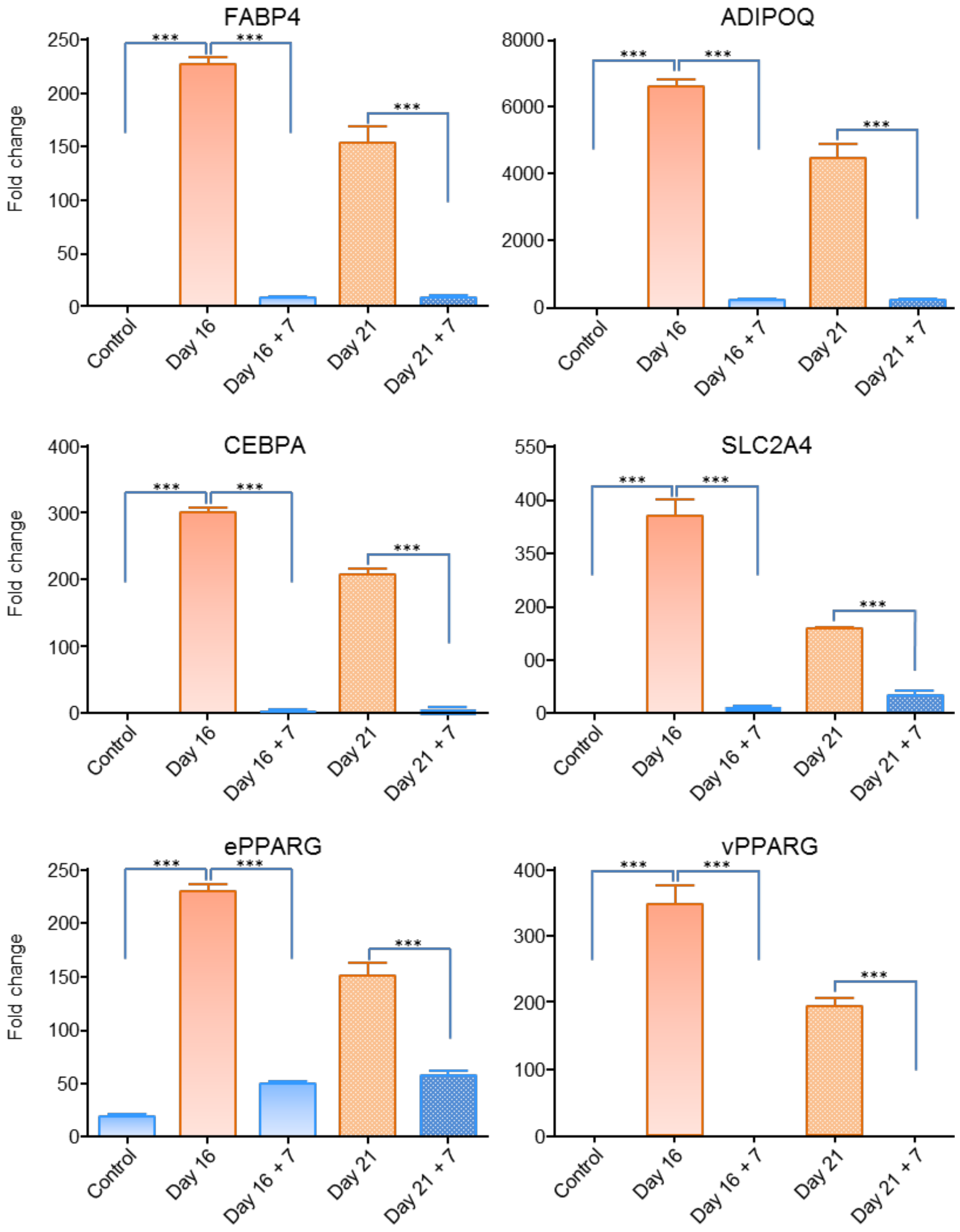

Supplementary Figure S7. Marked decrease in expression of adipocyte specific genes in mature differentiated adipocyte-like cells 7 days after withdraw of PPARγ2 overexpression inducing reagent DOX. Endogenous PPARγ (ePPARG) expression was determined by RT-PCR using a pair of primers specific to a 3'-UTR region immediately after PPARg stop codon while pSLIK-PPARγ2 expression was determined by RT-PCR using a forward primer that is specific to a vector sequence immediately before the PPARγ2 open reading frame and a reverse primer that is specific to a PPARγ2 open reading frame sequence at the 5' end. Data from three independent experiments are represented as mean ± SEM (***P<0.001, *t*-Test).

Disease Models & Mechanisms • Supplementary information

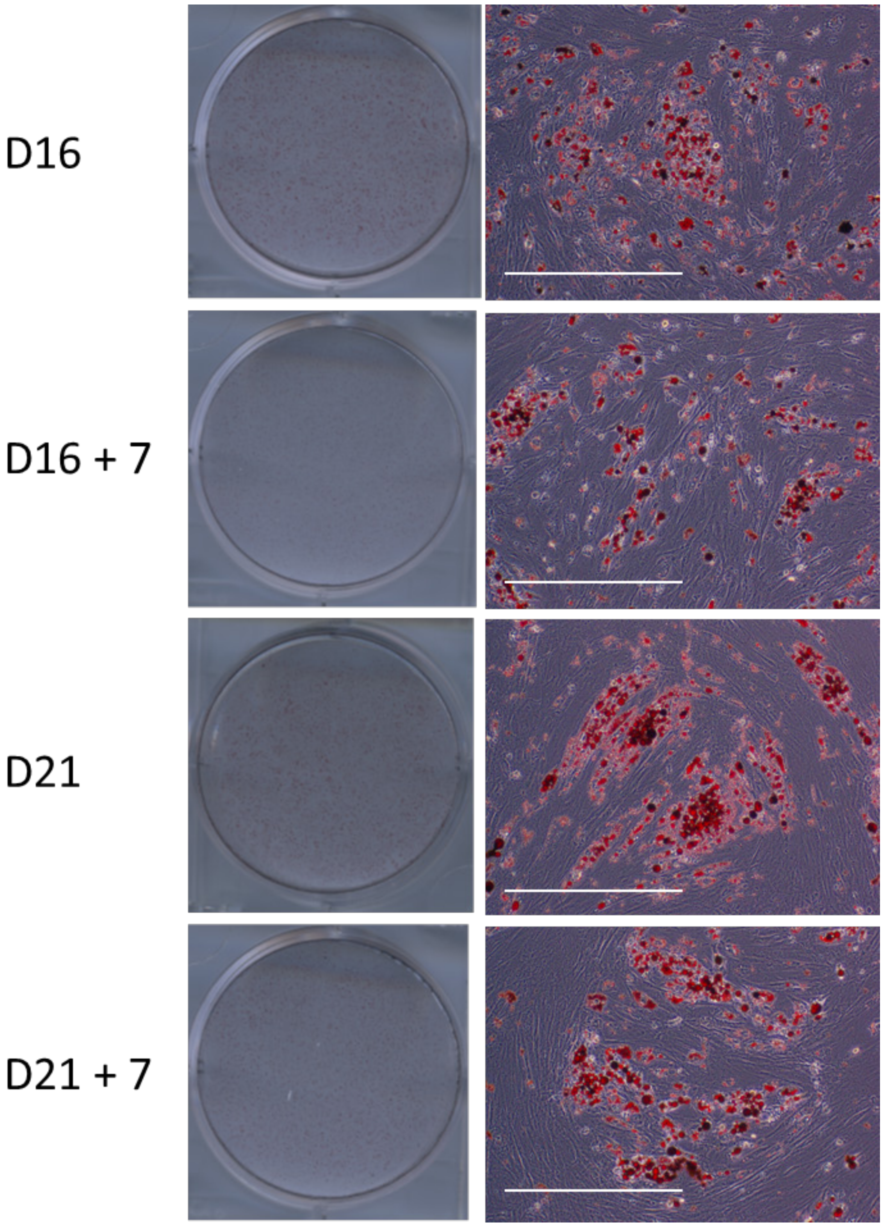

Supplementary Figure S8. Delipidation of mature differentiated adipocyte-like cells via pluripotent stem cell stage using protocol described in supplementary Figure S6 did not occur 7 days after withdraw of PPARγ2 overexpression inducing reagent DOX. (scale bar: 200 μm)

Disease Models & Mechanisms • Supplementary information

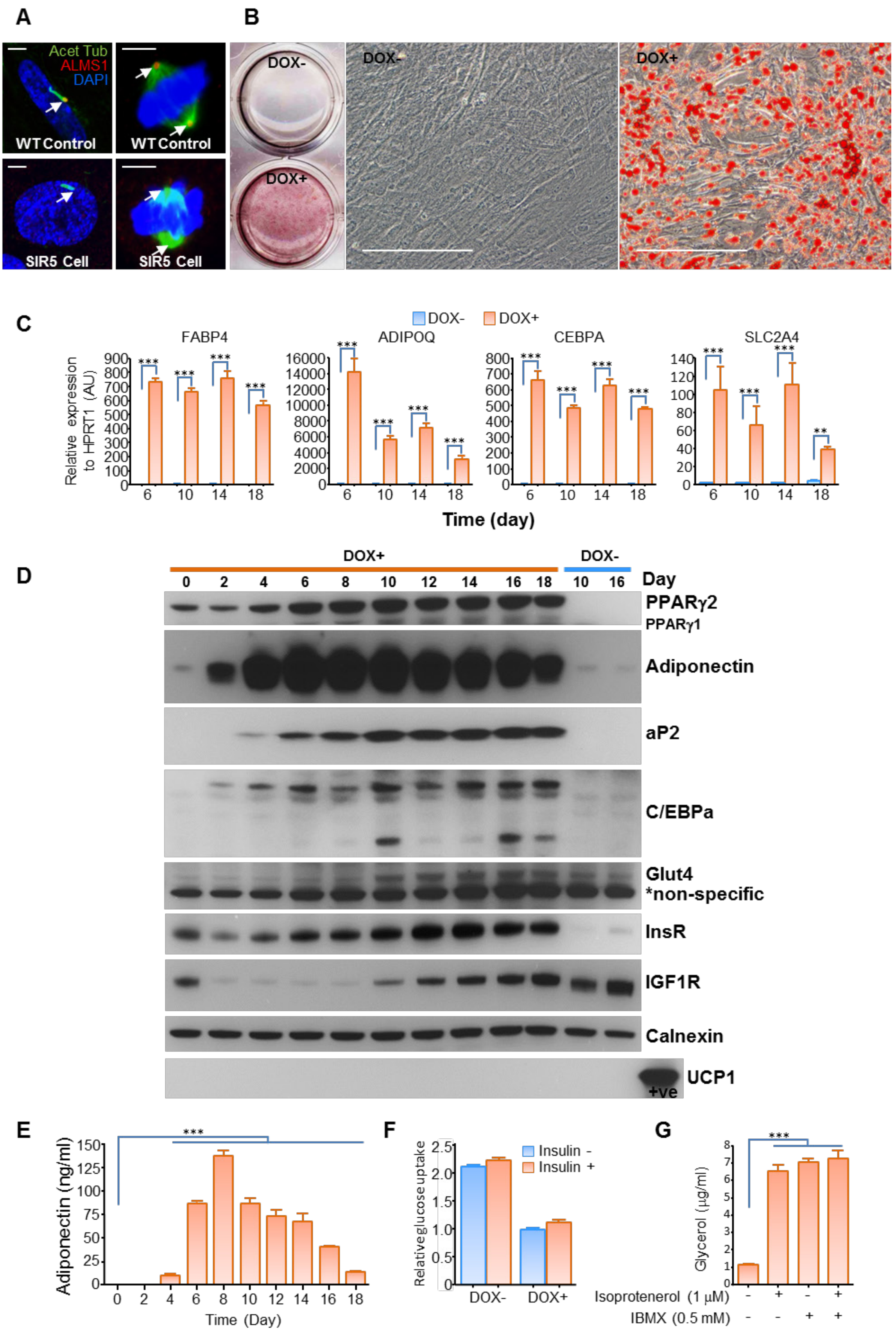

Disease Models & Mechanisms • Supplementary information

Supplementary Figure S9. Direct reprogramming of dermal fibroblasts derived from an Alström Syndrome patient. (A) Confocal immunofluorescence analysis revealed absence of ALMS1 at centrosome and the basal body of primary cilium in the patient cells due to the disease causing mutations on the *ALMS1* gene. (scale bar: 5 μm) (B) Oil Red O staining showing a successful conversion of ALMS1 dermal fibroblasts into adipocyte-like cells. (scale bar: 200 μm) (C) Quantitative RT-PCR analysis of expression of marker genes of white adipocytes at four different differentiation time points. (D) Western blot analysis of key proteins in adipocyte-like cells during direct reprogramming up to day 18, using antibodies as indicated. (E) Adiponectin secretion from direct reprogrammed adipocyte-likes cells. 48 hour culture media were determined with DELFIA. (F) Glucose uptake assay. (G) Lipolysis assay of direct reprogrammed adipocyte-like cells treated with isoprotenerol and/or IBMX. Data from three independent experiments are represented as mean ± SEM (**P<0.01, ***P<0.001, *t*-Test).

Disease Models & Mechanisms • Supplementary information

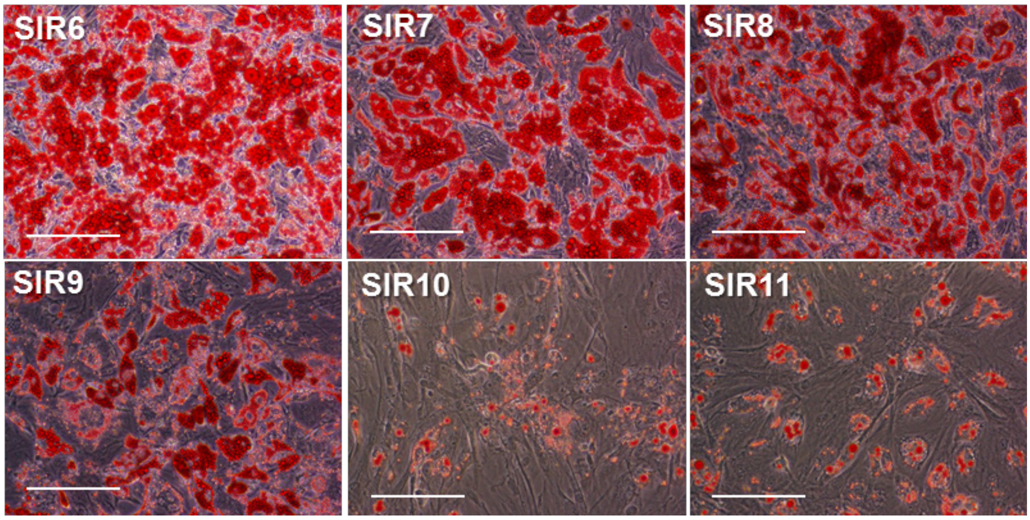

Supplementary Figure S10. Direct reprogramming of dermal fibroblasts derived from lipodystrophy patients harbouring mutations on *WRN, BLM, NSMCE2, PIK3R1 and PCYT1A* genes. (scale bar: 200 μm)

Supplementary References

Chen, J. H., T. Geberhiwot, et al. (2017). "Refining genotype-phenotype correlation in Alstrom syndrome through study of primary human fibroblasts." Mol Genet Genomic Med **5**(4): 390-404.

Huang-Doran, I., P. Tomlinson, et al. (2016). "Insulin resistance uncoupled from dyslipidemia due to C-terminal PIK3R1 mutations." JCI Insight **1**(17): e88766.

Kumar, S., P. N. Durrington, et al. (1996). "Severe insulin resistance, diabetes mellitus, hypertriglyceridemia, and pseudoacromegaly." J Clin Endocrinol Metab **81**(10): 3465-3468.

Payne, F., R. Colnaghi, et al. (2014). "Hypomorphism in human NSMCE2 linked to primordial dwarfism and insulin resistance." J Clin Invest **124**(9): 4028-4038.

Payne, F., K. Lim, et al. (2014). "Mutations disrupting the Kennedy phosphatidylcholine pathway in humans with congenital lipodystrophy and fatty liver disease." Proc Natl Acad Sci U S A **111**(24): 8901-8906.

Simpkin, A., E. Cochran, et al. (2014). "Insulin Receptor and the Kidney: Nephrocalcinosis in Patients with Recessive INSR Mutations." Nephron Physiol **128**: 55-61.

Disease Models & Mechanisms • Supplementary information
